# Supplementary material for: HIV-1 with gag processing defects activates cGAS sensing
Source: Retrovirology. 2024 May 23;21:10. doi: 10.1186/s12977-024-00643-0 (PMC11112816; doi:10.1186/s12977-024-00643-0)
Supplement: Supplementary file 1 — Supplementary material [file 12977_2024_643_MOESM1_ESM.docx]

Supplementary material


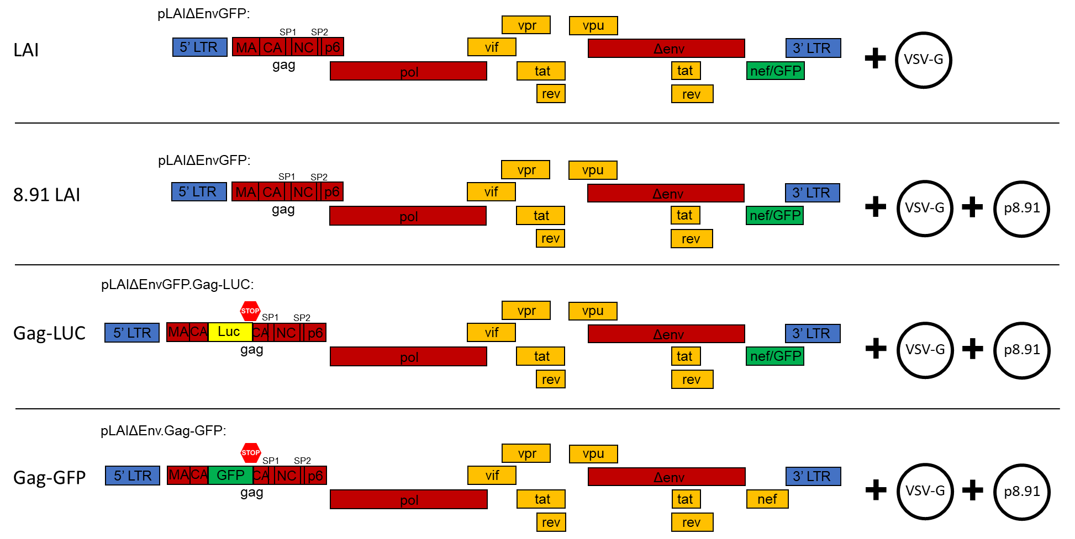


Suppl Fig 1: Schematic of wild-type and Gag-fusion viruses

Schematic representation of the plasmids transfected into HEK293T cells to produce WT and Gag-fusion viruses. The genome plasmid of each virus is based on the HIV-1 LAI strain[32] with a deletion in envelope (Δenv) and expressing GFP in the place of Nef (pLAIΔEnvGFP), except Gag-GFP. Each virus was pseudotyped with VSV-G, and for 8.91 LAI, Gag-LUC and Gag-GFP viruses, were co-transfected with p8.91 packaging construct encoding Gag-Pol, Tat and Rev[60]. LTR: long terminal repeat, MA: matrix, CA: capsid, SP: spacer peptide, NC: nucleocapsid, env: envelope, Luc: firefly luciferase, GFP: green fluorescent protein.


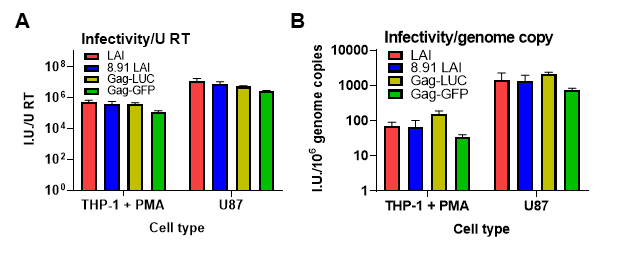


Suppl Fig 2: Particle infectivity of wild-type and Gag-fusion viruses

A: Virus infectivity (infectious units, I.U.) on THP-1 cells differentiated with PMA or U87 cells (measured by flow cytometry at 48 h post-transduction) normalised to units of RT (measured by SG-PERT).

B: Virus infectivity (infectious units, I.U.) on THP-1 cells differentiated with PMA or U87 cells (measured by flow cytometry at 48 h post-transduction) normalised to genome copy number (measured by qPCR).

Data are mean ± SD from biological triplicates of a single experiment, representative of at least 2 repeats.


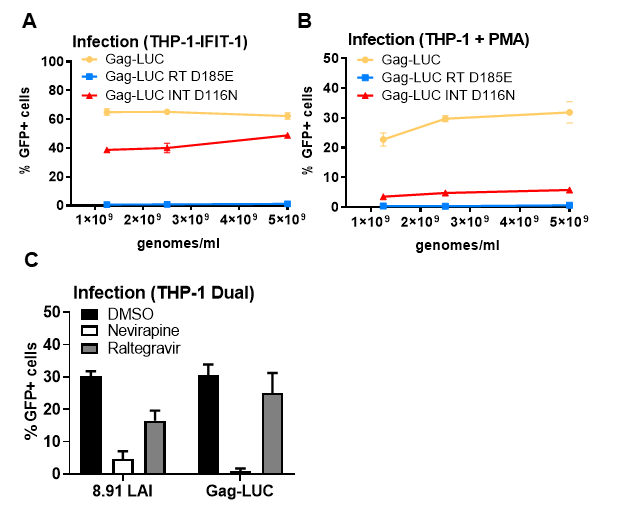


Suppl. Fig 3: ISG induction by HIV-1 Gag-fusion virus is RT-dependent

A: Infection data from Fig 3A. THP-1-IFIT-1 cells transduced for 48 h with Gag-LUC, RT-defective Gag-LUC (Gag-LUC RT D185E) or integrase-defective Gag-LUC (Gag-LUC INT D116N) at 1.25×10^9^, 2.5×10^9^ and 5×10^9^ genomes/ml.

B: Infection data from Fig 3B-D. PMA-treated THP-1 shSAMHD1 cells transduced for 48 h with Gag-LUC, Gag-LUC RT D185E or Gag-LUC INT D116N at 1.25×10^9^, 2.5×10^9^ and 5×10^9^ genomes/ml.

C: Infection data from Fig 3E-G. THP-1 Dual cells transduced for 48 h with 8.91 LAI or Gag-Luc (1.5 U RT/ml) in the presence of DMSO vehicle, 5 μM neviripine or 10 μM raltegravir.

Data are mean ± SD from biological triplicates of a single experiment, representative of at least 3 repeats.


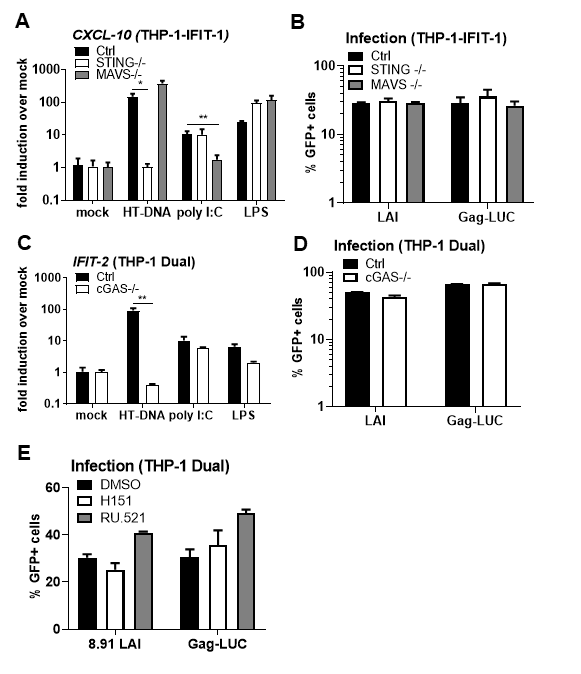


Suppl. Fig 4: ISG induction by HIV-1 Gag-fusion virus is dependent on cGAS and STING

A: *CXCL-10* ISG qPCR from monocytic THP-1-IFIT-1 cells lacking STING or MAVS, or a gRNA control (Ctrl) cell line stimulated for 24 h with 0.1 μg/ml HT-DNA, 0.5 μg/ml poly I:C or 50 ng/ml LPS.

B: Infection data from Fig 4A-C. THP-1-IFIT-1 cells lacking STING or MAVS, or a gRNA control (Ctrl) cell line transduced for 48 h with WT LAI or Gag-LUC (1.5 U RT/ml).

C: *IFIT-2* ISG qPCR from monocytic THP-1 Dual cells lacking cGAS, or a gRNA control (Ctrl) cell line stimulated for 24 h with 0.1 μg/ml HT-DNA, 0.5 μg/ml poly I:C or 50 ng/ml LPS.

D: Infection data from Fig 4D-F. THP-1 Dual cells lacking cGAS, or a gRNA control (Ctrl) cell line transduced for 48 h with WT LAI or Gag-LUC (1.5 U RT/ml).

E: Infection data from Fig 4G. THP-1 Dual cells lacking cGAS, or a gRNA control (Ctrl) cell line transduced for 48 h with WT LAI or Gag-LUC (1.5 U RT/ml) in the presence of DMSO vehicle, 0.5 μg/ml STING inhibitor H151 or 10 μg/ml cGAS inhibitor RU.521

Data are mean ± SD from biological triplicates of a single experiment, representative of at least 3 repeats. Statistical analyses were performed using Student's t‐test, with Welch's correction where appropriate, comparing to Ctrl cells as indicated. *P < 0.05, **P < 0.01.


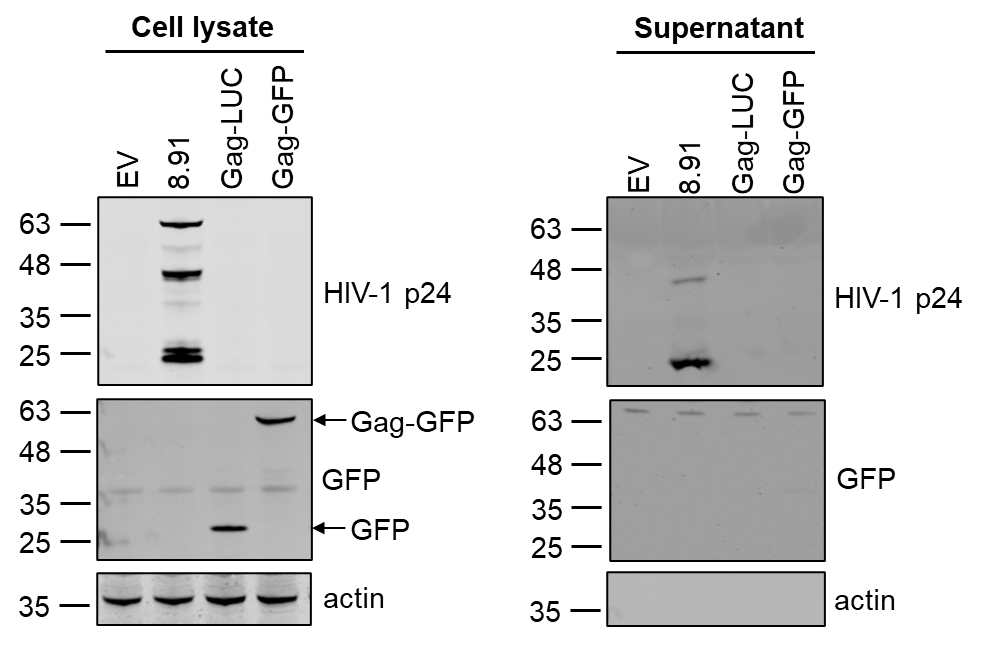


Suppl. Fig 5: Gag-fusion viruses display defects in Gag processing

Immunoblot of whole cell lysates or the supernatant from HEK293T cells transfected for 48 h with 8.91, Gag-LUC or Gag-GFP plasmids alone, or an empty vector (EV) as a control, detecting HIV-1 p24, GFP or actin. The Gag-GFP fusion (expressed from the Gag-GFP plasmid) and free GFP (expressed from the Gag-LUC plasmid) are indicated with arrows.

Suppl. Fig 6: Gag-fusion viruses have reduced capacity to saturate TRIM5α

Third replicate assay of data presented in Fig. 5B. FRhK4 cells were co-transduced with a fixed dose of WT LAI.GFP (5×10^7^ genomes/ml) and increasing doses of the WT/Gag-LUC chimeric viruses carrying a luciferase-expressing genome (0.0005 – 1 U RT/ml). Rescue of GFP infectivity was assessed by flow cytometry at 48 h. Data are singlet % GFP values.
